# In vitro secretion and activity profiles of matrix metalloproteinases, MMP-9 and MMP-2, in human term extra-placental membranes after exposure to *Escherichia coli*

**DOI:** 10.1186/1477-7827-9-13

**Published:** 2011-01-25

**Authors:** Veronica Zaga-Clavellina, Guadalupe Garcia-Lopez, Arturo Flores-Pliego, Horacio Merchant-Larios, Felipe Vadillo-Ortega

**Affiliations:** 1 Biomedical Research Branch, Instituto Nacional de Perinatologia "Isidro Espinosa de los Reyes", Mexico City, Mexico; 2 Direction of Research, Instituto Nacional de Perinatologia "Isidro Espinosa de los Reyes", Mexico City, Mexico; 3 Biomedical Research Institute, School of Medicine, Universidad Nacional Autónoma de México, Mexico City, Mexico; 4 Departament of Experimental Medicine, School of Medicine, Universidad Nacional Autónoma de México, Mexico City, Mexico

## Abstract

**Background:**

Premature rupture of fetal membranes (PROM) complicated with intrauterine infection has been associated to alterations of the extracellular matrix (ECM) homeostasis. The aim of this work was to evaluate the integral/functional response of the amnion (AMN) and choriodecidua (CHD) to synthesis, secretion, and activity of MMP-2 and MMP-9 and of their inhibitors TIMP-1, -2, and -4, after stimulation with *Escherichia coli*.

**Methods:**

Full-thickness membranes were mounted on a Transwell device, constituting two independent chambers, *Escherichia coli *(1×10 (6) CFU/mL) were added to either the amniotic or the choriodecidual face or to both. Secretion profiles of MMP-2, MMP-9, TIMP-1, TIMP-2, and TIMP-4 were quantified by ELISA and gelatinolytic activity by zymography. Immunoreactivity for MMP-2 and MMP-9 was revealed by immunohistochemistry and the collagen content was assessed by the hydroxyproline assay.

**Results:**

Levels of MMP-9 in CHD and AMN increased 4- and 8-fold, respectively, after simultaneous infection. MMP-2 secreted to the medium by CHD increased a mean of 3 times after direct stimulation. Secretion profiles of TIMP-1, TIMP-2, and TIMP-4 remained without significant changes. Collagen content was significantly decreased (4-fold) in infected membranes, and was associated with loss of structural continuity and co-localization with immunoreactive forms of MMP-2 and MMP-9.

**Conclusions:**

Infection of chorioamniotic membranes with *E. coli *induces an increase in the secretion of inactive forms and an association to ECM of active forms of MMP-2 and MMP-9 without changes in TIMP-1, -2, and -4. These changes could explain the significant decrease of collagen content and loss of structural continuity.

## Background

During human pregnancy, multiple strategies are displayed to keep the fetus in an immunologically privileged environment in the amniotic cavity delimited and surrounded by the extra-placental membranes [1,2]. This tissue is highly organized as a bi-laminated complex formed from fetal-derived amnion (AMN) and chorion, which, interdigitate with the maternal decidua and constitute the choriodecidua (CHD) layer. The integrity of all these cell types, in close association with their extracellular matrix (ECM), preserves the membrane's structure and tensile strength to warrant its role as a selective barrier between the fetal and maternal compartments [3].

The chorioamniotic ECM is composed of several different types of collagen arranged in a complex framework, maximizing its mechanical resistance. Major components are types I, III, IV, V, and VI collagens and abundant proteoglycans, which are embedded in the fibrous proteins. The principal tissue support is generated by fibers composed of types I and III collagens, which, in turn, are stabilized by a network of collagen types: IV, V, and VI [4,5].

A fine equilibrium between degradation and synthesis of the ECM has been ascribed to matrix metalloproteinases (MMPs) activity, a group of structurally related zinc-dependent enzyme endopeptidases, whose proteolytic activity is regulated via its interaction with specific tissue inhibitors of metalloproteinases (TIMPs).

There is experimental evidence indicating that an imbalance in the stoichiometric relation MMPs/TIMPs (1:1) could account for the increase in MMP activity associated to a microenvironment in which the ECM degradation rate is higher than the synthesis rate [6].

MMP-2 (72 kDa type IV collagenase [gelatinase A]) and MMP-9 (92 kDa type IV collagenase [gelatinase B]) have been ascribed roles as essential mediators in degradation/damage of the ECM of fetal membranes in normal conditions; in this scenario, the ECM loses its structure and mechanical strength at around labor onset synchronously with increased myometrial activity and cervical ripening, allowing delivery to occur [7-9].

The pathologic rupture of extra-placental membranes is known as premature rupture of the membranes (PROM), a condition in which rupture occurs before the onset of labor; this occurs in among 5 to 15% of all pregnancies and is directly associated with 25 to 50% of preterm births [3,10].

Epidemiological and clinical studies have identified a number of factors associated with increased risk for PROM; however, intrauterine infections caused by bacteria are considered a predominant cause/risk factor. Once the pathogen reaches the uterine cavity through an ascending route, the extra placental membrane is the last barrier these pathogens need to break before accessing the amniotic cavity [11-13].

In this pathological scenario, the rupture of the chorioamnion results from a degrading process that is potentiated by an exacerbated immunological response [14-17]. Previous evidence generated at our laboratory indicates that stimulation of human fetal membranes with *S. agalactiae, *a microorganism with high prevalence in cases of vaginal/intrauterine infections, is followed by active secretion of MMP-2 and MMP-9 [16].

The present study was undertaken to evaluate the integral/functional response and individual contribution of the AMN and CHD to the secretion and activity of MMP-2 and MMP-9 after stimulation with *Escherichia coli*, a highly pathogenic gram-negative bacterium that has been associated with preterm delivery and PROM in humans [11], as well as pregnancy losses [18,19] and neurological injury in preterm infants [20]; and which inoculated endocervically in pregnant rabbits elicits a histologic inflammation in the maternal and fetal compartments [21].

To achieve these goals, we used an *ex vivo *culture system in which human term chorioamniotic membranes were mounted on Transwell devices, physically separating the upper and lower chamber. This model allowed us to reproduce the differential contact between the AMN/CHD region and the pathogen, and to know the source and secretion patterns on both sides of the membranes [15].

## Methods

Ten pregnant women (37-40 weeks of gestation), 22-35 years old, being under the care of the Obstetrics Outpatient Service at the Instituto Nacional de Perinatologia "Isidro Espinosa de los Reyes" in Mexico City were recruited for this study. All women had uneventful pregnancies, without evidence of active labor and with neither clinical nor microbiological signs of chorioamnionitis/lower genital tract infection.

All women provided written informed consent immediately after delivery by elective cesarean section and before collecting samples. The protocol and the collection and use of the samples were approved by the Institutional Review Board.

General microbiological analyses, included aerobic and anaerobic microorganisms, were conducted on the placenta and extra-placental membranes immediately after delivery, only membranes with proved sterility were used for this study.

### Fetal membrane explants culture

After delivery, the extra-placental membranes were cut at a distance of 4 to 6 cm from the placental disc, transported to the laboratory in sterile Dulbecco Modified Eagle Medium (DMEM; Gibco BRL, Bethesda, MD), and rinsed in sterile saline solution (0.9% NaCl) to remove adherent blood clots.

Explants of 18 mm diameter were manually cut into 18 mm diameter discs and held together with silicone rubber rings to be placed on the upper chamber of a Transwell system (CORNING, New York, NY) from which the original polycarbonate membrane had been removed previously. This experimental model has been validated and published previously [15-17], and allows us to test two independent compartments delimited by the AMN and CHD of the fetal membrane.

One milliliter of DMEM supplemented with 10% fetal bovine serum FBS (DMEM-FBS) (Gibco BRL), 1 mM sodium pyruvate, and 1X antibiotic-antimycotic solution (penicillin 100 U/mL, streptomycin 100 μg/mL) were added to each chamber. The mounted explants were placed in a 12-well tissue culture plate (CORNING, New York, NY) and incubated in 5% CO_2 _at 37°C.

### Explants stimulation

To stabilize tissues after manipulation, the extra-placental membrane explants were pre-incubated for 24 h in the DMEM-FBS medium. Subsequently, the explants were washed with saline solution (0.9% NaCl) to remove FBS and the remainder medium was changed to DMEM supplemented with 0.2% lactalbumin hydrolysate (Gibco BRL), 1 mM sodium pyruvate, and 1X antibiotic-antimycotic solution (penicillin 100 U/mL, streptomycin 100 μg/mL), and co-incubated with 1 × 10^6 ^colony forming units (CFU) of *Escherichia coli *isolated from a cervicovaginal exudate from a patient with PROM (35 weeks) and intrauterine infection diagnosis.

The inoculum size of 1 × 10^6 ^CFU has been previously standardized/published by our group to induce secretion of different anti-inflammatory cytokines in human fetal membranes, as well as by Davies and cols., in a rabbit model of acute intra-amniotic infection [21].

Additionally, in a recent study, Grigsby and cols. demonstrated that inoculation, via an indwelling catheter, of the chorion/decidua and myometrium with 1 × 10^6 ^CFU of Group B Streptococci, another microorganism associated with preterm labor and PROM, is the only effective way to induce clinical signs of preterm labor in a nonhuman primate model [22].

Four Each experiment (n = 10) included the following set of chambers, in triplicate: Basal, control membranes in which only the medium culture was added to the compartments; CHD, *E. coli *was added only to the choriodecidua side; AMN, *E. coli *was added only to the amniotic compartment; Both, the bacterium was added simultaneously to both compartments.

To know if the incubation with TIMPs can revert/prevent the degrading effect of MMPs induced by the stimulation with *E. coli*, an extra set of chambers was included in which a mix of TIMP1 and TIMP2 (500 ng/mL) was added at the same time (Calbiochem, Darmstadt, Germany).

After 24 h of co-incubation, the medium from the AMN and CHD chambers was collected and centrifuged at 5000 rpm, for 3 min at 4°C, to remove bacteria. Bacteria-free supernatant of each sample was stored at -80°C until assayed. Protein concentration in all samples was measured according to the Bradford method [23].

Tissues from each single treatment and control were processed for structural analysis, tissue lysates, hydroxyproline assay, and immunohystrochemistry.

### Structural analysis

Extra-placental membrane fragments from control and stimulated conditions were fixed in Karnovski buffer, pH 7.3 (0.1 mM sodium cacodylate, 2.5% glutaraldehyde) (Sigma Chemical Co, St Louis, MO), for 2 h at room temperature. Samples were dehydrated by standard techniques and embedded in EPON (Pelco, Glovis, CA). Slides with 1-μm sections were stained with 0.5% toluidine blue (Sigma) and analyzed through light microscopy.

### Tissue lysate

After stimulation with *Escherichia coli*, the membranes were washed three times with cold PBS; subsequently, the tissues were exposed to a thermal shock (4°C for 10 min, 60°C for 3 min, -20°C for 10 min, and 37°C for 5 min) in buffer A (50 mM Tris Base, 100 mM NaCl, 20 mM CaCl_2_, 0.02% NaN_3_, and 2% Triton 100X). After these incubations, tissues were homogenized in 1 mL of buffer B (50 mM Tris Base, 100 mM NaCl, 40 mM EDTA, 0.02% NaN_3_). The soluble fraction was obtained after centrifugation at 14 000 rpm, 20 min; followed by quantification using the Bradford method [23].

### Hydroxyproline assay

After treatment, 20 mg of each explant was rinsed with PBS to remove remnants of culture medium and bacteria, the explants were then hydrolyzed in 6 N HCl, at 110°C in dark for 18 h. After this period, samples were washed three times with distilled water to remove the acid. Conversion of hydroxyproline to pyrrole-2-carboxylic acid was achieved by incubating with 0.25 M of chloramine T (Sigma) in ethylene glycol monomethyl ether, for 20 min at room temperature.

After oxidation, the samples were vigorously vortexed with toluene, the colorimetric determination was done in the organic phase, an aliquot of each sample was mixed with Ehrlich's reactive (ethanol containing 2.0 M *p*-dimethylaminobenzaldehyde and 6.5% v/v of sulfuric acid); the colorimetric reaction was read at 560 nm.

### Zymography

SDS-polyacrylamide gels were co-polymerized with porcine gelatin (1 mg/mL) and loaded with either culture medium (0.5 μg/well) or tissue (6 μg/well) in non-denaturing loading buffer. Electrophoresis was performed under constant current (10 mA) for 1.5 h at 4°C. Gels were washed in 2.5% Triton X-100 for 30 min to eliminate SDS remnants and incubated overnight at 37°C in an activation buffer (50 mM Tris, pH 7.4, 0.15 M NaCl, 20 mM CaCl_2_, and 0.02% NaN_3)_

Gels were stained with 0.1% R-250 brilliant blue (Sigma). MMP-2 and MMP-9, constitutively secreted by U-937 (ATCC; Rockville, MD), a promyelocyte cell line, was used as activity standard marker. Concentrations of protein loaded onto the gels were applied within linearity of enzymatic activity; quantitation and comparison of the gelatinolytic activity (relative intensity of lysis bands) of MMP-9 and MMP-2 were performed by densitometry analysis with the NIH-Image software program V 1.6 (NIH, Bethesda, MD).

### MMP-2 and MMP-9 enzyme-linked immunosorbent assay in culture medium

Commercial kits (Amersham Biosciences, Buckinghamshire, UK) were used to quantify total MMP-9 and MMP-2 (zymogen and active forms) concentrations in the culture medium. Experimental procedures followed manufacturer's instructions. Briefly, proteins were captured onto the wells by incubating at 4°C for 18 h in a 96-well plate pre-coated with a specific antibody. After washing, the plates were incubated at 37°C with the substrate protein, after 4 h the resulting reaction was read at 405 nm using an ELISA plate reader. MMP-9 was measured in the range of 1 to 32 ng/mL with a sensitivity of 0.6 ng/mL. The MMP-2 standard curve was 1.5 to 24 ng/mL with a sensitivity of 0.37 ng/mL.

### Pro-enzyme and active form of MMP-9 ELISA in tissue lysates

To quantify the pro-enzymatic and active forms of MMP-9 in samples of tissue lysates a commercial kit (Amersham Biosciences) was used. This system is based on the detection of endogenous active MMP-9; quantitation for total enzyme (active and zymogen) was done by artificial activation with APMA (p-aminphenylmercuric acetate). Proteins were captured on the wells by incubating at 4°C, for 18 h in a 96-well plate pre-coated with a specific antibody. After washing, the plates were incubated at 37°C with the substrate protein and, after 4 h, the resulting reaction was read at 405 nm using an ELISA plate reader. MMP-9 was measured in the range of 0.5-16 ng/mL with a sensitivity of 0.5 ng/mL.

### TIMP-1, TIMP-2, and TIMP-4 enzyme-linked immunosorbent assay

Commercial kits were used for the quantitative determination of TIMP-1, TIMP-2 (Amersham Biosciences, and TIMP-4 (R&D Systems, Minneapolis, MN).

Experimental procedures followed manufacturer's instructions. The assays are based on a two-site enzyme-linked immunosorbent assay (ELISA) sandwich format, which uses two antibodies directed against different epitopes. Samples and standards were incubated per duplicate in the plates pre-coated with specific antibodies for 2 h at room temperature. The plates were then washed and incubated with horseradish peroxidase-conjugated antibody to form an immobilized complex. The resulting color was read at 405 nm for TIMP-1 and TIMP-2 and at 450 nm for TIMP-4. TIMP1 was measured in the range of 3.13 to 50 ng/mL with a sensitivity of 1.25 ng/mL; TIMP2 standard curve was from 8 to 18 ng/mL with a sensitivity of 3 ng/mL, and TIMP-4 standard curve was from 0.078 to 5 ng/mL with a sensitivity of 0.002 ng/mL.

### Immunohistochemistry

After stimulation with *E.coli*, sections of 10 to 15 μm of paraffin-embedded extra-placental membranes were processed for immunohistochemical staining using mouse mAbs to MMP-9 (IgG1, Clone 56-2A4) or MMP-2 ( IgG1,Clone 42-5D11) (Calbiochem, Darmstadt, Germany). Both antibodies were used at a 1:50 dilution, membranes without stimulation were used as control. Binding of primary antibodies was detected using the avidin-biotinylated peroxidase technique and biotinylated horse anti-mouse IgG (Vector, Burlingame, CA). Tissue sections were counterstained with Mayer's hematoxylin and cover-slipped for evaluation by light microscopy.

### Statistical analysis

Analysis was performed with the Sigma Stat 2.03 (Jandel Scientific Software, Chicago, IL). Statistical significance was determined by one-way analysis of variance. Tukey's test was used to assign individual differences. Where the data failed a normality test, significance was determined using a Kruskal-Wallis test. *P *< 0.05 was regarded as significant.

## Results

### Secretion pattern of MMP-2 and MMP-9 to the culture medium

**Figure 1:**
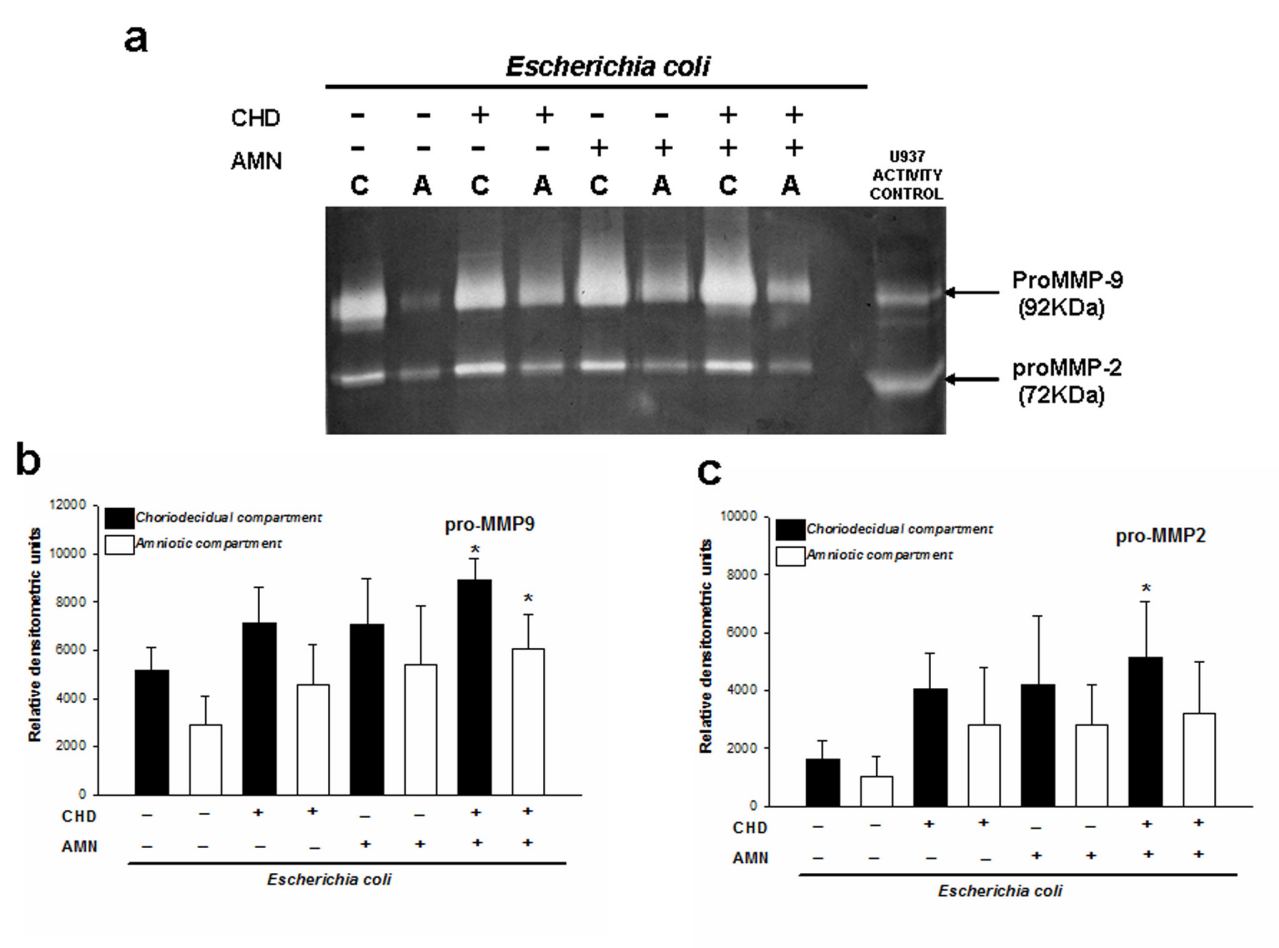
**Secretion patterns of pro-MMP-9 and pro-MMP-2 to culture medium**. (**a**) Representative gelatin-gel zymography showing enzymatic activities of proMMP-9 and proMMP-2 secreted to the culture medium by fetal membranes after selective infection with 1 × 10^6 ^CFU of *E. coli*. Each lysis band induced by pro-MMP-9 (**b**) and pro-MMP-2 (**c**) was quantified by densitometric analysis. Equal amounts of protein were loaded (0.5 μg). Each bar represents the mean and standard deviation of nine independent experiments; significant differences between basal and stimulated condition are indicated (*P < 0.05).

Basal secretion of pro-MMP-2 and pro-MMP-9 to the culture medium was increased after *E. coli *stimulation (Figure 1a); zymogram densitometric analysis showed that, regardless of the initial side of infection, the CHD and AMN secreted higher amounts of the zymogen form of both gelatinases in comparison with the basal conditions. However, the increase was significant only when the stimulus was applied on both sides at the same time, that is, CHD and AMM secreted 2.0-fold more MMP-9. The increase in MMP-2 secretion was significant in the choriodecidual compartment only after simultaneous stimulation, and zymogen was 3-times higher than in the basal condition (Figure 1b, c).

**Figure 2:**
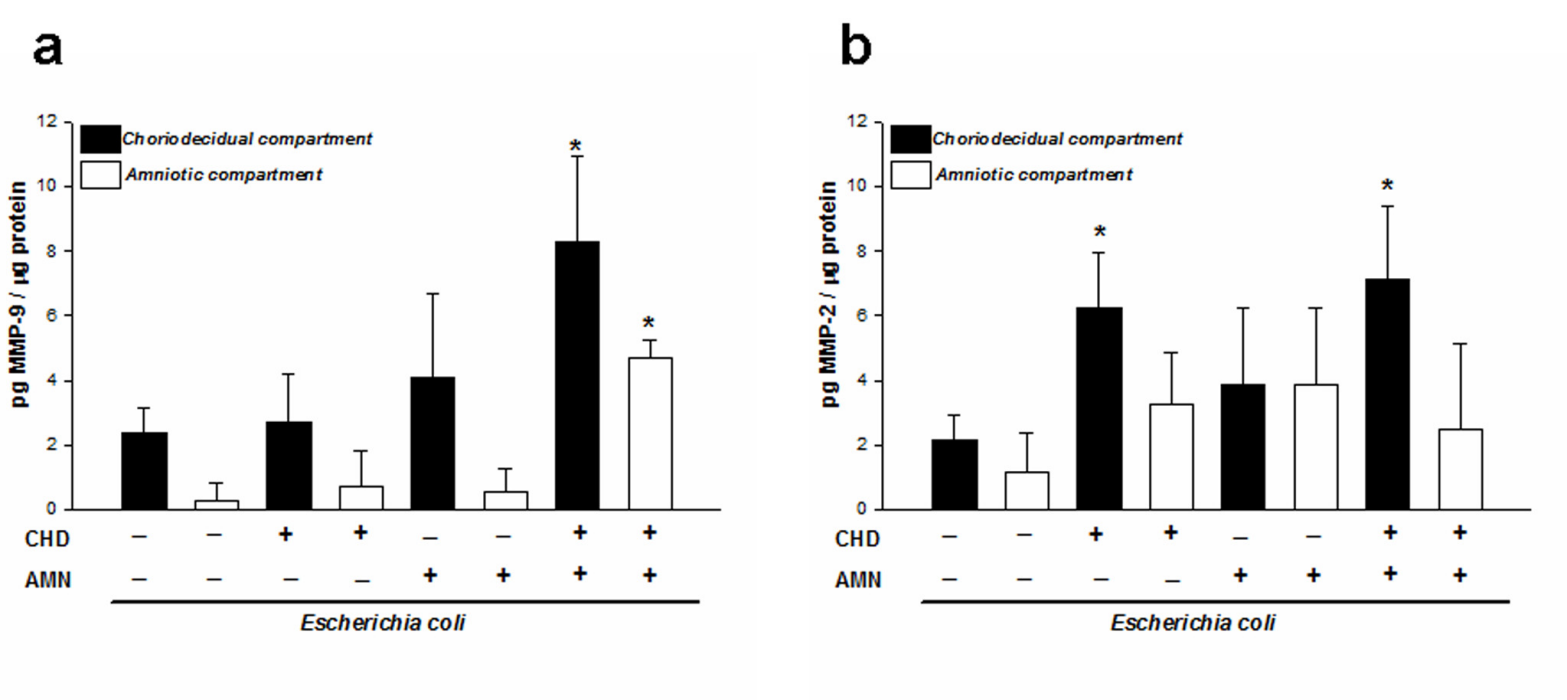
**Compartmentalized *in vitro *secretion of total MMP-9 and MMP-2**. Quantitation by ELISA of MMP-9 (**a**) and MMP-2 (**b**) in amnion (AMN) and choriodecidua (CHD) regions after selective stimulation with *E. coli.* Each bar represents the mean and standard deviation of nine independent experiments; significant differences between basal and stimulated condition are indicated (*P < 0.05).

The zymogen and active form of MMP-2 and MMP-9 were quantitated in the culture medium with specific ELISA; these assays confirmed that, when the stimulus was applied on both sides, MMP-9 increased 4-fold in the CHD region, whereas in the AMN the increase was 8-fold with respect to the respective controls (Figure 2a). MMP-2 secretion profile showed that although its concentration was increased in the AMN side with the three infection modalities, this increase was not significant. On the other hand, secretion in the CHD increased significantly (6.30 ± 1.13 pg/mL) (*P *≤ 0.05) only when this region was stimulated directly (Figure 2b).

### Secretion pattern of TIMPs to the culture medium

Regarding the effect of stimulation with *E. coli *on TIMPs, ELISA assays indicated that TIMP-1, TIMP-2, and TIMP-4 concentrations were not significantly different (*P *= 0.31, 0.42, and 0.56, respectively) from those found in basal conditions (Table 1).

**Table 1:** In vitro secretion of TIMP-1, TIMP-2 and TIMP-4 after selective exposure with 1X106 UFC of E. coli Interquartile range 50 (25-75)

*Zone of exposure to *E. coli	*TIMP-1 (ng/μg protein)*	*TIMP-2 (pg/μg protein)*	*TIMP-4 (pg/μg protein)*
**Choriodecidua**			
*Basal*	**9.56** (5.77-20.61)	**1199.57**(817.46-2882.14)	**4.00** (2.90-4.00)
*Both*	**16.05** (12.92-24.58)	**1332.31** (796.21-2284.64)	**4.00** (3.24-4.00)
*Choriodecidua*	**11.18** (5.88-26.52)	**1223.05** (822.56-3825.59)	**3.29** (1.08-2.4.00)
*Amnion*	**19.62** (5.58-29.47)	**1151.41** (739.56-2613.44)	**3.30** (2.17-4.00)
**Amnion**			
*Basal*	**5.44** (2.55-9.20)	**788.04** (616.23-1248.48)	**3.02** (0.31-3.66)
*Both*	**5.73** (4.45-10.07)	**776.13** (581.57-1408.88)	**1.88** (0.67-4.00)
*Choriodecidua*	**7.47** (5.43-21.27)	**1273.24** (752.46-2984.43)	**1.93** (1.30-4.00)
*Amnion*	**5.80** (2.79-11.61)	**843.23** (434.1-1578.75)	**4.00** (2.84-4.00)

Interquartile range 50 (25-75)

### Structural analysis of human chorioamniotic membranes

**Figure 3:**
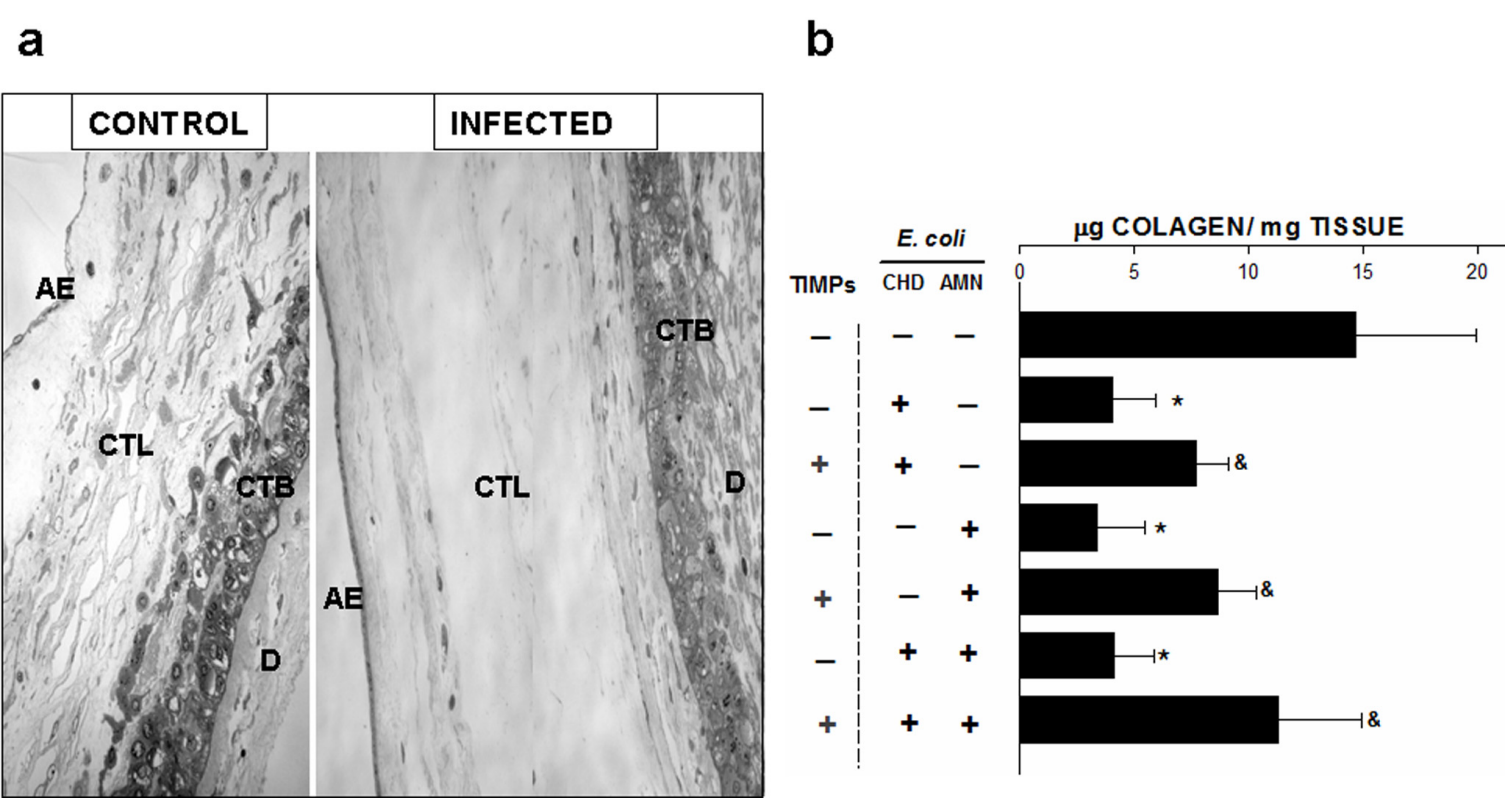
**Histologic analysis and collagen content of human chorioamniotic membranes**. (**a**) Histologic analysis of membranes control and infected during 24 h with *E. coli *in the amnion (AMN) and the choriodecidua (CHD) simultaneously, showing that the reticular structure of the amnion has been extensively degraded and hydrated, producing loss of structural continuity between both regions. AE, amniotic epithelium; CTL, connective tissue layer; CTB, cytotrophoblasts; D, decidua. Original magnification 20X. (**b**) Collagen content in membranes from three different stimulation modalities with their corresponding groups, in which the explants were incubated simultaneously with the TIMPs. Each bar represents the mean and standard deviation of eight independent experiments. Significant differences between basal and stimulated conditions are indicated (*P < 0.05). Groups simultaneously treated with the bacterium and TIMPs were compared with their respective group stimulated only with the bacterium (&*P *< 0.05).

The structural analysis of samples revealed a clear process of degradation in membranes treated with *E coli*. Compact and intermediate (spongy) layers showed a strong and extensive ECM degradation process with a discontinuous appearance and the tissular structure was clearly altered; these results were similar in samples from the three stimulation modalities (Figure 3a).

### Collagen content

A set of explants from each stimulation modality, including those that were co-stimulated with TIMP's, was used to determine the collagen content. The results show that, in comparison with basal conditions (14.66 ± 5.27 μg collagen/mg tissue), the collagen concentration in the explants that were differentially stimulated with *E. coli *decreased to a mean of 4.23 ± 1.9 μg/mg tissue (Figure 3b), which represents a 71% decrease. On the other hand, in those explants that were co-stimulated with TIMPs, the collagen content was in average 9.67 ± 2.66 μg collagen/mg tissue, which represents a recovery of 37% as compared to the groups that were stimulated only with the bacterium (Figure 3b).

**Figure 4:**
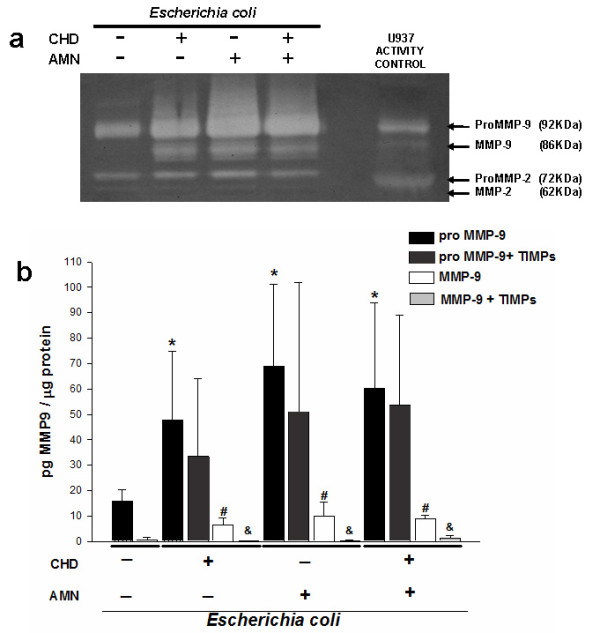
**MMP-2 and MMP-9 associated to tissue**. (**a**) Representative zymogram showing enzymatic activity of tissue extracts of membranes differentially stimulated with 1 × 10^6 ^CFU of *E. coli*. Equal amounts of protein were loaded (6.0 μg). (**b)** Tissular content of zymogen and active form of MMP-9 after selective stimulation with the bacterium during 24 h. A group was included in which the membranes were incubated simultaneously with 500 ng/mL of TIMP1 and TIMP-2; proMMP-9 + TIMPs group was compared with its respective pro-MMP-9 group; on the other hand, MMP-9 + TIMPs group was compared with its respective MMP-9 group. The graphic data show the mean ± SD and * # &*P *< 0.05 was considered significant.

A constant finding under all experimental conditions was that there was no increase in the secretion of active MMP-9 and MMP-2 into the culture medium. In order to characterize the possible presence of active forms of both gelatinases in close association with the tissue, tissue lysates were loaded on the zymogram, revealing an increased presence and activity of the active forms, mainly of MMP-9 (Figure 4a).

In order to obtain quantitative data to support these findings, a specific ELISA was performed using the tissue lysates to characterize the profile of active and zymogen forms of MMP9. The results show that, compared with the basal conditions, the pro-MMP9 and MMP9 level increased to a mean of 58.95 ± 10.71 pg/μg protein and 8.43 ± 1.79 pg/μg protein, respectively.

Regarding the groups of explants co-stimulated with the TIMPs and compared with their respective groups stimulated only with bacterium enabled us to see that although there was a diminution of the level of pro-MMP9 (46.0 ± 10.90 pg/μg protein), this change was not statistically significant; however, in comparison with the groups of explants that were differentially stimulated with *E. coli*, the level of active MMP-9 in the tissue was decreased significantly [0.58 ± 0.60 pg/μg protein] in those membranes that were co-cultured with TIMP1 and TIMP2 (Figure 4b).

### Immunohistochemistry

**Figure 5:**
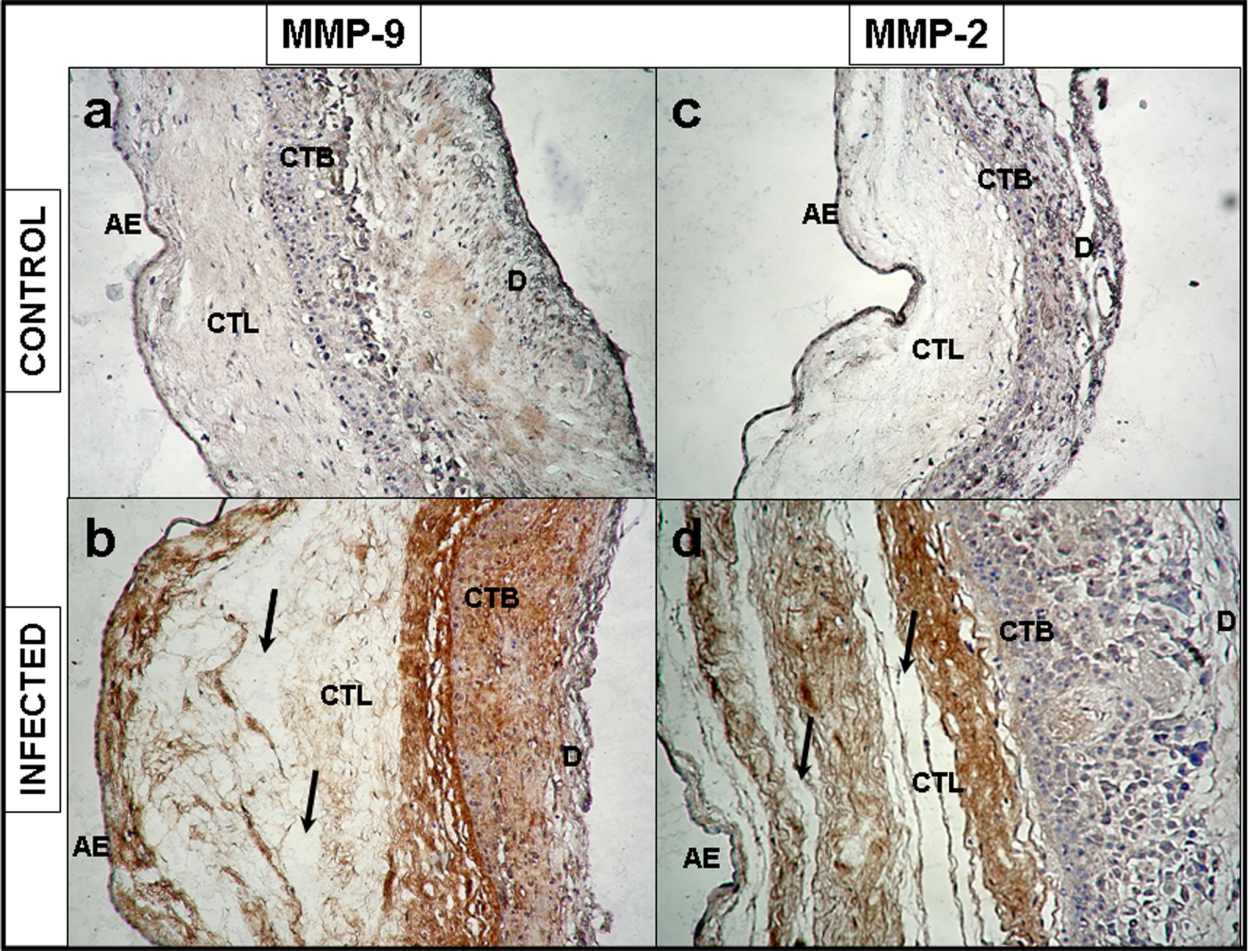
**Immunoreactivity of MMP-9 and MMP-2 in human chorioamniotic membranes**. Immunolocalization of MMP-9 (**a,b**) and MMP-2 (**c,d**) membranes after simultaneous stimulation with 1 × 10^6 ^CFU of *E. coli *during 24 h. AE, amniotic epithelium; CTL, connective tissue layer; CTB, cytotrophoblasts; D, decidua. Original magnification 20X.

The immunohistochemistry of membranes stimulated on both sides simultaneously showed a strong MMP-9 immunoreactivity in the amniotic epithelium, basement membrane, compact and intermediate layers of AMN, as well as in the reticular layer, basement membrane, and trophoblast cells of CHD (Figure 5a, b)

Immunoreactivity profile of MMP-2 showed a positive signal in the amniotic epithelium, basement membrane, compact layer, and intermediate layers, but this signal was less strong in trophoblasts (Figure 5c, d).

## Discussion

The results obtained indicate that stimulation of human term extra-placental membranes with *E. coli *induces an increase in the secretion and activity of MMP-2 and MMP-9, mainly by the CHD region, as well as a significant decrease in collagen content.

Previous studies by ourselves and others have demonstrated that intra-uterine/intra-amniotic infection, caused by the pathogens ascending from the cervix-vaginal region [16], as well as endotoxins [17], fosters an imbalance between MMPs and TIMPs and, consequently, increases enzymatic activity, favoring degradation and destruction of different elements of the ECM in extra-placental membranes [24-27].

In the present study, we used *Escherichia coli*, a gram negative bacterium that has been associated with harmful effects, such as the uncontrolled up-regulation of pro-inflammatory cytokines during human pregnancy [11,12,28,29], which have been reproduced in different animal models [30,31].

The experimental design used herein allowed us to emulate three different clinical stages of intrauterine ascending infection in human term extra-placental membranes: (i) choriodecidual infection, in which bacteria reach the CHD through ascendant colonization from the lower urogenital tract (corresponding to CHD's *E. coli*-stimulation); (ii) intra-amniotic infection, in which the infectious stimulus gains access to the amniotic cavity, a condition that could happen under iatrogenic introduction of microorganisms during invasive procedures such as amniocentesis (corresponding to AMN's *E. coli*-stimulation); and (iii) chorioamniotic infection, a situation in which bacteria come in contact with both sides of the membranes at late stages of colonization, which implies that bacteria have crossed the membranes, from the choriodecidual region toward the intra-amniotic cavity.

The results demonstrate that, regardless of which side of the membrane was the first zone of contact with *E. coli*, the co-culture with the bacterium induced increase of pro-MMP2 and pro-MMP9 levels secreted to the culture medium mainly by the choriodecidual region; however, when the stimulus was applied simultaneously on both sides of the membrane, the secretion of both zymogens was significant in both regions.

In order to obtain quantitative data to support these findings, we quantified by ELISA total MMP2 and MMP9 (zymogen and active form), which proved that, in terms of total enzyme, it was the simultaneous stimulation which induced the main secretion of both gelatinases to the culture medium.

The presence in the culture medium of both gelatinases only in their pro-enzymatic form did not coincide with the extensive degradation of the ECM, mainly in compact and intermediate (spongy) layers of the amnion. In biological terms, degradation of the ECM should be attributed to the active enzyme; there is evidence indicating that the active forms of MMP-9 and MMP-2 have a higher affinity for their substrate [32], for this reason we assume that the most immunoreactive forms in the tissue are active forms.

Our results demonstrate the presence of active forms of MMP-9 in tissue lysates from membranes infected with *E. coli*. Activated MMP-9 has been ascribed a role as central mediator in degradation/damage of the extracellular matrix of term extra-placental membranes in normal and pathological conditions [7,8,33,34]. The increase of this enzyme in the maternal-fetal unit has been correlated with the signals of labor onset during preterm pregnancies and preterm premature rupture of membranes (PPROM) [35].

There is evidence indicating that decreased collagen content [36], altered collagen structure, and increased collagenolytic activity [37] are characteristic of fetal membranes that rupture prematurely. Our results demonstrate that, regardless of the modality of stimulation with *E. coli*, collagen content of term extra-placental membranes decreased markedly.

To demonstrate, that the degradation of collagen is associated, at least in part, with the activity of MMP-9, a set of explants was incubated with TIMP-1 and TIMP-2, both inhibitors of MMP-9. Results showed that in comparison with the explants incubated only with the bacterium, the explants that were co-incubated with TIMP-1 and TIMP-2 had significant less degradation of collagen, which agreed with the significant diminution of the active MMP9 form level.

Additionally, specific MMP-9 immunoreactivity was revealed after selective stimulation with *E. coli*; immunohistochemistry showed that zones with high type IV collagen content, such as basement membrane and compact layer, were positive to MMP-9.

On the other hand the stimulation with *E. coli *produced a significant increase of proMMP-2, which was corroborated by ELISA and zymography; this zymogen was secreted to the culture medium principally by the choriodecidua region. However, the increase of the zymogen form was not translated into a significant increase in the level of the active form, as evidenced by characterizing the gelatinolytic activity pattern of tissue lysates from infected membranes.

Our results are in agreement with previous clinical and experimental evidence indicating that the expression of the active form of MMP-2 does not change in extra-placental membranes and amniotic fluid during human term parturition with intact membranes and PROM [38,39].

The secretion of pro-MMP-2 to the culture medium and non-significant changes in the concentration of the active form have been previously reported in human chorioamniotic membranes in culture [16] and in an intrauterine infection model using primates [14] infected with *S. agalactiae*

On the other hand, the secretion profile of TIMP-1,TIMP-2, and TIMP-3 did not change significantly under any infection modality; this result contrasts with previous evidence indicating that stimulating chorioamniotic membranes with LPS induces a significant decrease of TIMP-2 [24,25,40,41], but which agrees with evidence demonstrating that intrauterine infection in pregnant rhesus monkeys induces a significant diminution of TIMP-1 [16].

Previous evidence indicate that selective stimulation of chorioamniotic membranes with *E. coli *[29] and LPS [15] increases in a differential and significant manner the level of IL-1β, TNF-α, IL-6, and IL-8, mainly in the choriodecidua region; these soluble factors are potentially harmful to pregnancy, because their synthesis and secretion are magnified and all factors in the signal network, including prostaglandins and MMP's, are also up-regulated [42].

The present experimental model was designed to test the individual contributions of amnion and choriodecidua from term extra-placental membranes, and although this two compartment tissue culture system mimics more closely the separation of fetal and maternal compartments *in vivo*, our results can only be focused on term pregnancies.

During physiological and pathological conditions the response of extra-placental membranes against infection is part of a very complex network of signals involving both, maternal and fetal, tissues. The onset of term labor associated with intrauterine infection can be considered a mixture of a mechanism of host defense against intrauterine infection, whereby the mother eliminates infected tissues and provides a survival asset that allows the fetus to escape a hostile intrauterine environment.

## Conclusions

The present work provides experimental evidence indicating that the differential stimulation of the term extra-placental membranes with *E. coli *is associated with the increase in the secretion/activity of MMP-2 and MMP-9, which significantly decreases collagen content, leading to severe ultrastructural changes in the tissue.

The increase in the concentration of zymogen and active forms of these gelatinases without changes in the TIMPs could explain part of the physiopathogenic mechanism that operates during an infectious process along term pregnancies.

## Abbreviations

AMN: Amnion; CHD: Choriodecidua; PROM: Premature rupture of membranes; ECM: Extra cellular matrix; MMPs: Metalloproteinases; TIMPs: Tissue inhibitors of metalloproteinases; IL-1β: Interleukin-1 beta; TNFα: Tumor necrosis factor alpha; IL-10: Interleukin-10; IL-6: Interleukin-6; GBS: group B streptococci; LPS: Lipopolysaccharide; TLR: Toll like receptors; ELISA: Enzyme-linked immunosorbent assay; DMEM: Dulbecco Modified Eagle medium; FBS: Fetal bovine serum; CFU: Colony forming unit.

## Competing interests

The authors declare that they have no competing interests.

## Authors' contributions

VZC, GGL, and AFP collected the samples, performed the microbiological control, culture membranes, stimulation with the bacterium, and ELISA assays. AFP performed the hydroxiproline assay. HML performed the structural analysis. VZC, HML, and FVO participated in the design of the study, data collection, and analysis, as well as manuscript preparation. All authors have read and approved the final manuscript.

## Acknoweledgements

This work was supported by the National Council of Science and Technology of Mexico (CONACyT) Grant No. 61590 to VZC. We thank Irma Elena Sosa-Gonzalez from the Infectology and Immunology Department for providing all the microbiological methodology and Jose Baltazar-Garcia, Pedro Medina and Pedro Flores from the National University Autonomous of Mexico (UNAM) for tissue processing.
